# Supplementary material for: Engineered caspases directly rewire mutant Ras to cell death
Source: bioRxiv. 2026 Sep 3:2026.08.06.743376. Preprint. [Version 2] doi: 10.64898/2026.08.06.743376 (PMC13684547; doi:10.64898/2026.08.06.743376)
Supplement: Supplementary file 1 [file NIHPP2026.08.06.743376v2-supplement-1.pdf]

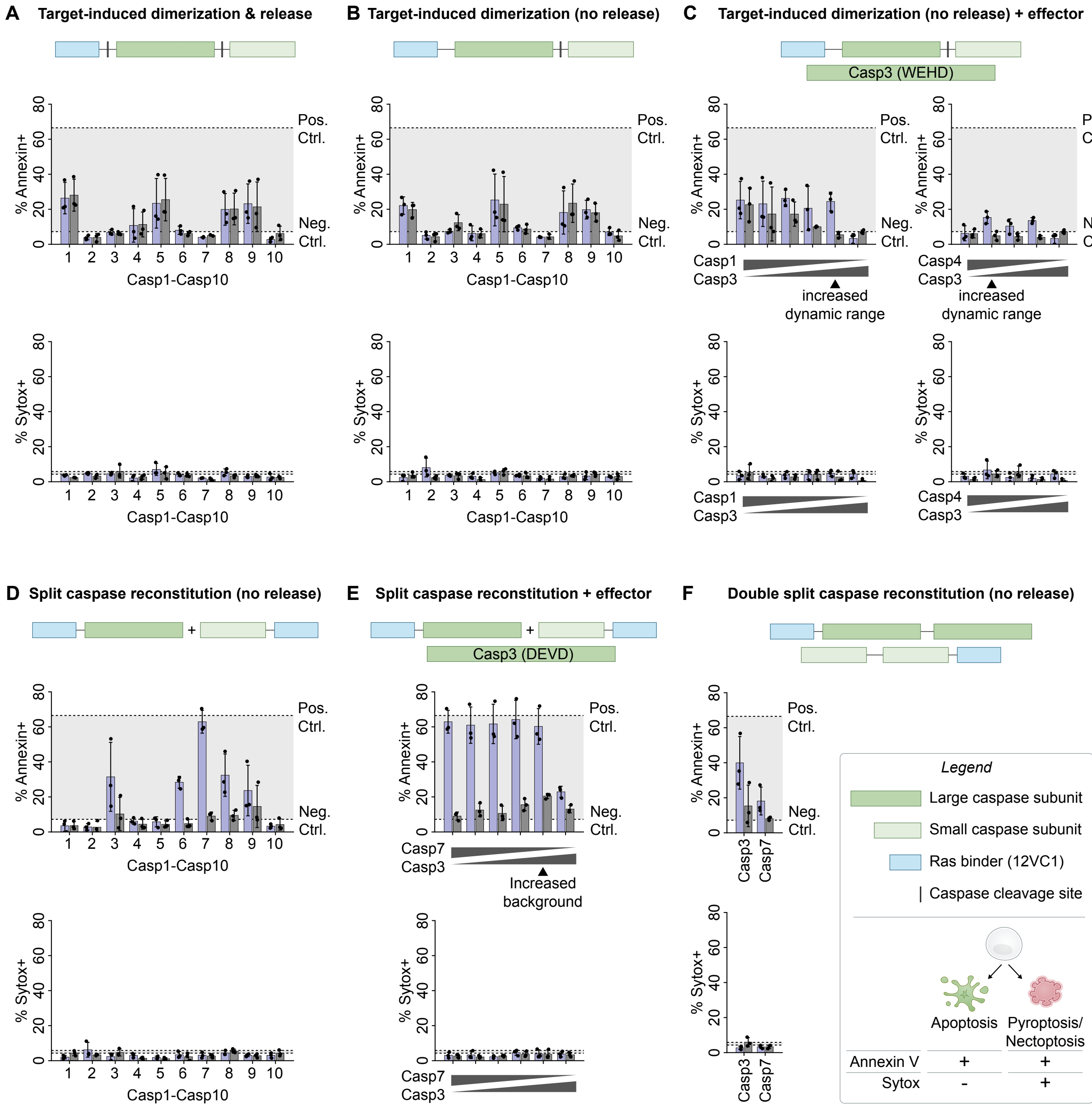

Figure S1. Overview of synthetic caspase designs tested for Ras targeting. (A)-(F) Purple and gray bars represent mean cell death (fraction of Annexin- or Sytox-positive cells) for $KRAS^{G12C}$ and $KRAS^{WT}$, respectively. Error bars indicate s.d. across replicates. **(C), (E)** Sensor-caspase plasmid amounts (left to right): 30, 25, 20, 15, 10 (except for Casp4), and 0 ng. Ectopic effector-caspase plasmid amounts (left to right): 0, 5, 10, 15, 20 (except for Casp4), and 30 ng.

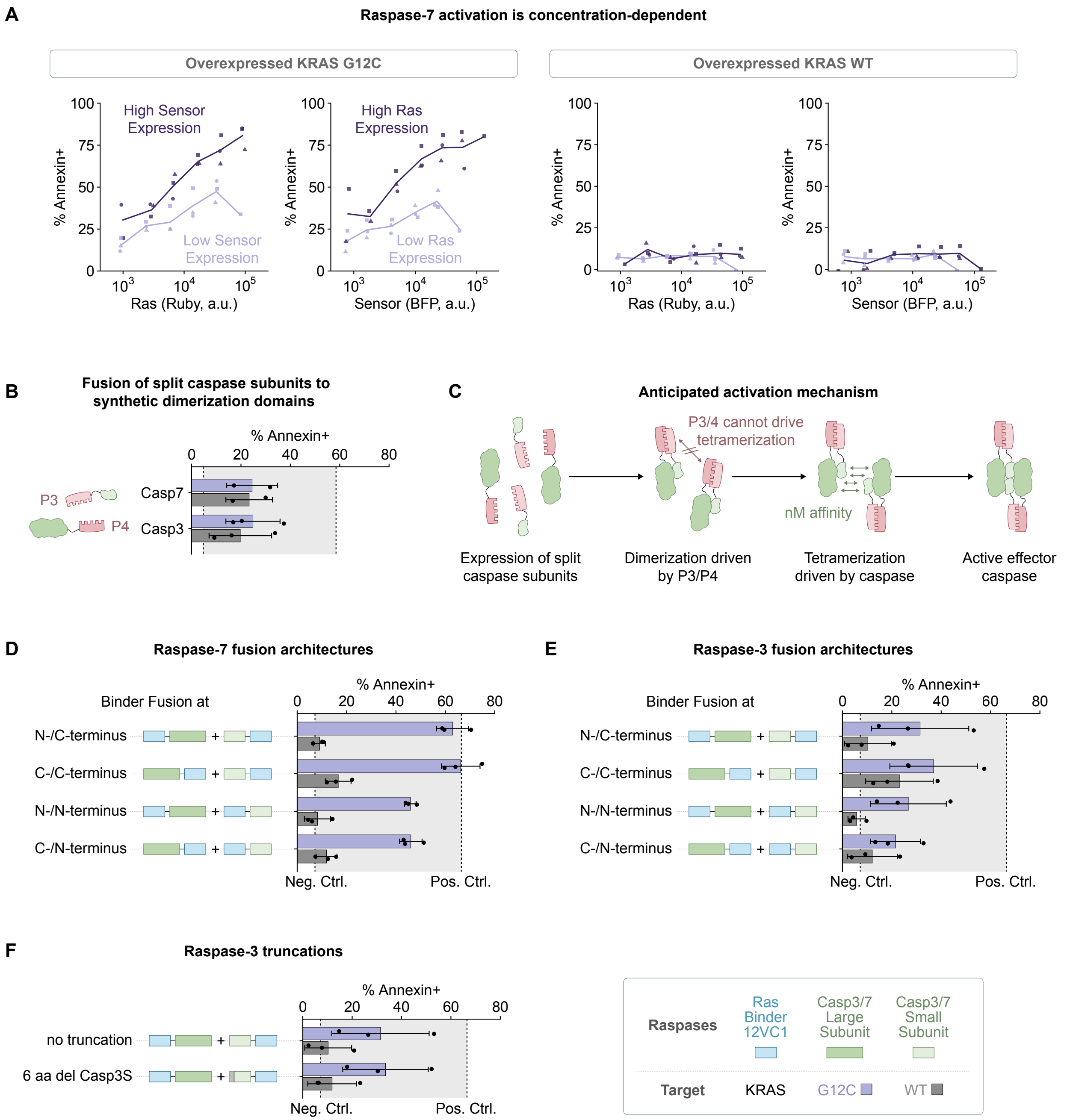

Figure S2. Mechanistic characterization of Raspase-7. **(A)** Raspase-7 activation depends on both Ras abundance and circuit expression. Ruby and BFP fluorescence were used to quantify Ras and caspase-sensor expression, respectively. Data points were generated by binning Ruby and BFP signals into discrete expression ranges; marker shapes are matched across replicates. **(B)** Cell death induced by constructs in which the P3/P4 dimerization domains were fused to complementary caspase subunits. Flow-cytometry gating: Ruby > 10^3 and 10^4 < BFP < $10^{4.5}$. Purple and gray bars report mean cell death (fraction Annexin V-positive cells) for $KRAS^{G12C}$ and $KRAS^{WT}$, respectively; error bars denote s.d. across replicates. **(C)** Proposed activation mechanism of split caspases: Dimerization of split caspase subunits is sufficient for sensor activation and cell death induction. **(D)-(F)** Raspases recapitulate key mechanistic features of native caspase activation: Purple and gray bars represent mean cell death for $KRAS^{G12C}$ and $KRAS^{WT}$, respectively; error bars indicate s.d. across replicates. **(D), (E)** Comparison of fusion architectures for Raspase-7 **(D)** and Raspase-3 **(E)**. **(F)** Evaluation of truncated circuit variants for Raspase-3.

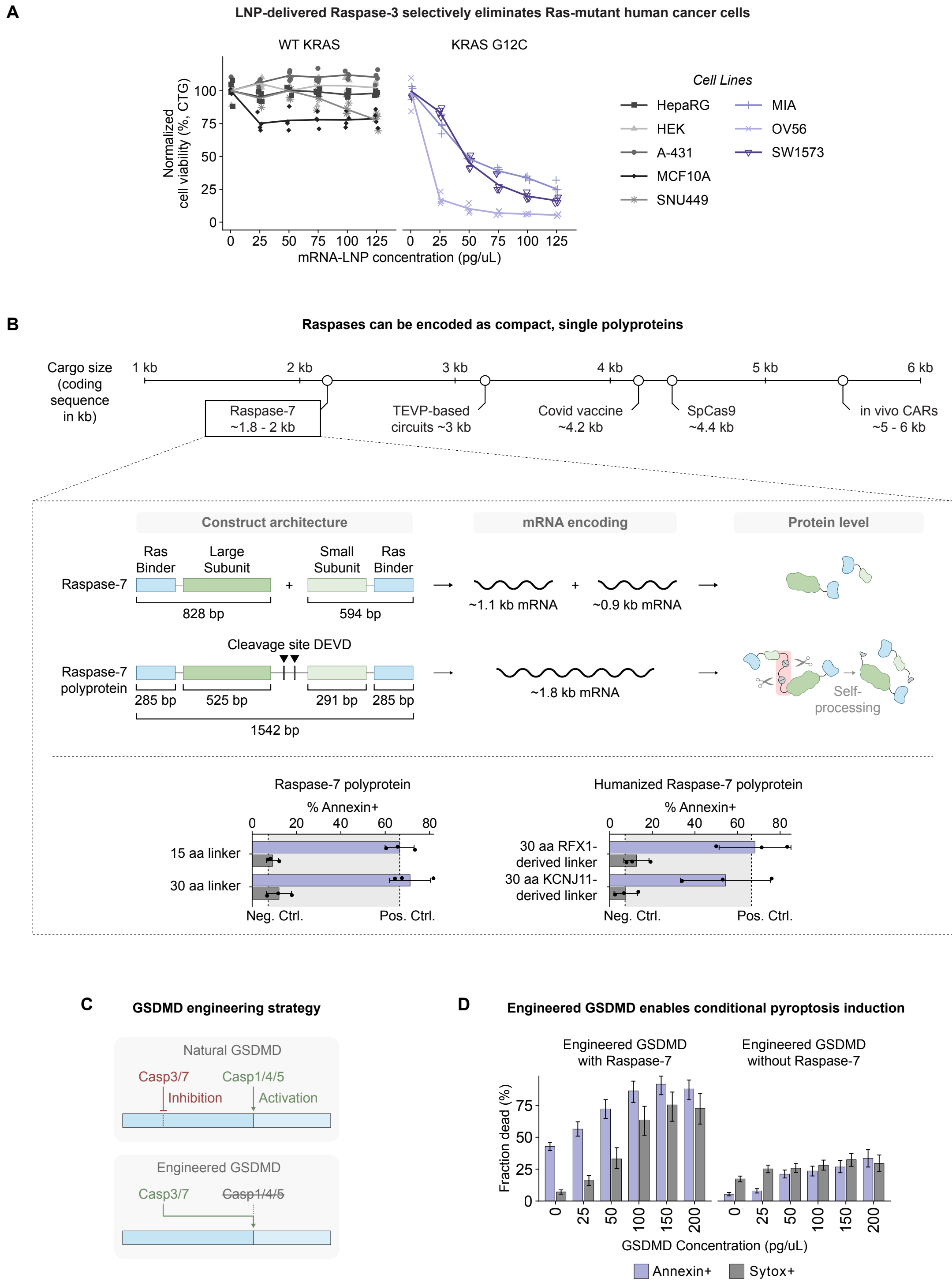

Figure S3. Raspases could have therapeutic utility. (A) LNP-delivered Raspase-3 selectively eliminates Ras-mutant cancer cells. Cell viability was measured 3d after circuit treatment by CellTiter-Glo and normalized to a mock LNP transfection control encoding NeoR. Points denote replicates; lines indicate the replicate mean. **(B)** Raspases have a compact cargo footprint that can be further reduced by expressing the circuit as a single polyprotein. Self-processing is the anticipated mechanism, but was not experimentally validated here. Purple and gray bars report mean cell death for $KRAS^{G12C}$ and $KRAS^{WT}$, respectively; error bars denote s.d. across replicates. **(C)** Raspases can be coupled to gasdermins to induce pyroptosis: Schematic of the GSDMD engineering strategy. **(D)** Fraction of Annexin V-positive and Sytox-positive cells following delivery of engineered GSDMD-supplemented Raspase-7. MIA PaCa-2 cells received either 150 pg/µL sensor (left) or 150 pg/µL NeoR (right) formulated as mRNA-LNP, together with 150 pg/µL H2B-mCherry mRNA as a co-transfection marker. Flow-cytometry gating: mCherry > 10^3. Bars denote means; error bars indicate bootstrap-derived 95% confidence intervals.

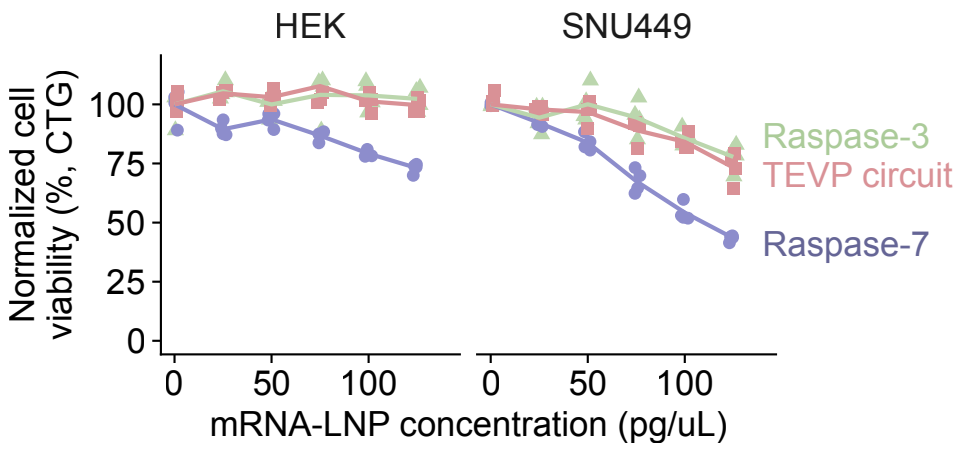

Figure S4. Comparison of Raspases and TEV protease-based Ras-targeting circuits in wild-type Ras cells. Viability of $KRAS^{WT}$ cells was measured by CellTiter-Glo 3d after treatment and normalized to the NeoR mock transfection control. Points denote replicates; lines indicate the replicate mean.
